# Association between employment status and self-rated health: Korean working conditions survey

**DOI:** 10.1186/s40557-016-0126-z

**Published:** 2016-09-08

**Authors:** Kimin Kwon, Jae Bum Park, Kyung-Jong Lee, Yoon-Sik Cho

**Affiliations:** Department of Occupational and Environmental Medicine, Ajou University Hospital, Suwon, South Korea

**Keywords:** Precarious work, Employment status, Self-rated health, Korean Working Conditions Survey

## Abstract

**Background:**

This research was conducted with an aim of determining the association between employment status and self-rated health.

**Methods:**

Using the data from the Third Korean Working Conditions Survey conducted in 2011, We included data from 34,783 respondents, excluding employers, self-employed workers, unpaid family workers, others. Self-rated health was compared according to employment status and a logistic regression analysis was performed.

**Results:**

Among the 34,783 workers, the number of permanent and non-permanent workers was 27,564 (79.2 %) and 7,219 (20.8 %). The risk that the self-rated health of non-permanent workers was poor was 1.20 times higher when both socio-demographic factors, work environment and work hazards were corrected.

**Conclusions:**

In this study, perceived health was found to be worse in the non-permanent workers than permanent workers. Additional research should investigate whether other factors mediate the relationship between employment status and perceived health.

## Background

With the global emergence of policies emphasizing labor flexibility, such as globalization and neoliberalism, which started several decades ago, the number of non-permanent workers has increased [1]. In Korea, following the 1997 economic crisis the labor structure changed with greater flexibility in the labor market, and this effect continues to this day. In this regard, employment status in the labor market show diverse patterns. In particular, non-permanent positions are increasing, and so too is the ratio of non-permanent workers to permanent workers, which means that non-permanent positions have come to play an important role in the labor market [2]. According to a report by Statistics Korea, the percentage of non-permanent workers among all paid employees was 35.5 % as of the third quarter of 2014. The reported conversion rate of non-permanent workers to permanent workers was 22.4 % and the hourly wage of non-permanent workers was just 64 % of that of permanent workers, which is markedly lower than other Organization for Economic Co-operation and Development member countries [3]. An increase in the number of non-permanent workers causes a variety of problems and research on this topic is being actively conducted.

One study on several occupational groups reported that unstable employment negatively affects perceived health status [4]. Non-permanent work is accompanied by job insecurity, income inequality, and the sense of loss in job stability and the associated anxiety negatively affects health. Unstable employment plays a role in reducing social safety and stability, and causes problems related to time, standing within the workplace, welfare, and low wages.

However, according to epidemiological investigations published to date, the effects of unstable employment on health have not been consistent. The results of studies reporting harmful health effects of unstable employment show that transition from a permanent position to an non-permanent position causes job insecurity and anxiety, which not only negatively affects an individual’s marriage, work motivation, and psychological health, such as worries, depression, and stress, but also has a large effect on family and group health [5–7]. Furthermore, compared to permanent positions, non-permanent positions are associated with a harmful work environment, such as simple and repetitive tasks, and sociopsychologically disadvantageous work characteristics [8]. Most non-permanent jobs have poorer work conditions, and long work hours and overtime work are known to be associated with deterioration in subjective health status and sociopsychological health [9]. Non-permanent work decreases job satisfaction, increases health risks due to drinking or smoking, and affects mortality due to various cancers and causes. In a study conducted in Finland, the mortality rate of non-permanent workers was 1.2–1.6 higher than that of permanent employees [2].

However, other cross-sectional studies did not report an association between non-permanent employment and negative health effects [10–12].

Currently, the number of non-permanent employees is increasing in Korea, and therefore it is necessary to investigate the actual difference in health status due to non-permanent work. However, the majority of studies to date were conducted in Europe or North America, and reports on unstable employment and accompanying health indicators in Korea are lacking. This is likely because there have not yet been many investigations on work environment and its effects on health, and there is no formalized questionnaire to determine health status.

We assessed self-rated health, reasoning that subjective health is determined by the environment and socio-statistical characteristics and that it could be considered a comprehensive health indicator by previous studies [13–17]. Therefore, based on data from the third Korean Working Conditions Survey, measured using a structured questionnaire tool on workers nationwide and we aimed to understand the reason.

## Methods

### Study population

This study used data from the 2011 Korean Working Conditions Survey, the third survey of the Occupational Safety and Health Research Institute (previous surveys were conducted in 2006 and 2010). Data were collected on employees older than 15 years of age living in 16 cities or provinces (including Jeju Island) in the Republic of Korea. Trained interviewers collected data from each respondent. A total of 50,032 respondents met the criteria and were classified as employers, self-employed workers, paid workers, unpaid family workers, or others. We included data from 34,783 respondents, excluding employers, self-employed workers, unpaid family workers, others, and 5 respondents for whom there was incomplete data.

### Subjects

Gender, age, educational level, monthly household income, alcohol, and smoking were chosen as the variables of the socio-demographic characteristics.

Classification of employment status in this study was based on response to the question in the Korean Working Conditions Survey "What is your position at your workplace among the following?" Those who responded as "1. Permanent worker" were defined as permanent employees, and those who responded as "2. Temporary worker" or "3. Daily worker" were defined as non-permanent workers.

Subjective health status was assessed by self-rated health and was based on response to the question "How is your health in general? Would you say it is…" Those who responded as "Very good" or "Good" were defined as having good subjective health status and those who responded as "Fair" or “Bad” or "Very bad" were defined as having poor subjective health status.

Subjects were divided into non-drinkers, those who drank the moderate amount of 1–2 glasses of soju less than 1–2 times a week, and those who drink more than that. Subjects were divided into non-smokers, those who smoked in the past but did not at the time of questioning, and current smokers.

Using the Korean Standard Classification of Occupations, occupations were classified as follows: white-collar jobs: professional technical jobs, senior management jobs, and office jobs; pink-collar jobs: sales jobs and service jobs; and blue-collar jobs: skilled jobs, semi-skilled jobs, unskilled jobs, and agriculture and forestry jobs [15].

In terms of the work environment, we analyzed work hours, shift work, occupational group, tenure, Job satisfaction, and Job instability. Work hours were classified according to the Labor Standards Act, with 52 hours used as the standard, including 40 legal working hours and 12 overtime work hours. Weekly work hours were divided into less than 40 hours, more than 40 but less than 52 hours, and more than 52 hours. Those who responded 'Yes' to the question "I am a shift worker' were defined as shift workers and those who answered 'No' were defined as non-shift workers. Tenure was classified into less than 1 year, between 1 and 10 years, and more than 10 years. Job satisfaction was identified through the question is satisfied in the working environment. Job instability was identified through the ‘I, within the next six months, would be to lose this job’ question.

Work hazards were categorized as physical, ergonomic hazards. Respondents were asked how often they are exposed to physical, chemical, biological, and ergonomic factors while working. Exposures to ergonomic factors were collected from one of seven choices as follows: (1) throughout the entire work, (2) throughout nearly all of the work, (3) throughout three-fourths of the work, (4) throughout half of the work, (5) throughout one-fourth of the work, (6) hardly exposed, or (7) never exposed. The respondents who answered either answers one through five, as listed above, were regarded as being exposed to work hazards. Physical hazards were exposures to vibrations from hand tools or machinery; noises that cause people to communicate in a very loud voice. Ergonomic hazards were being exposed to a tiring or painful posture, having to lift or move people, having to lift or move heavy things, standing or walking for extended periods, or performing repetitive hand or arm movements.

### Data analysis

We analyzed the difference in the socio-demographic characteristics and work environment between permanent and non-permanent employees as well as the difference in the socio-demographic characteristics and work environment according to self-rated health. After an adjustment for socio-demographic characteristics, working conditions, and working environments, a logistic regression analysis was conducted to determine if any socio-demographic characteristics, working conditions, or working environments were associated with self-rated health. The survey weighting was carried out on the basis of the economically active population. All analyses were performed using SPSS for Windows version 19.0 (SPSS Inc., Chicago, IL, USA).

## Results

### General characteristics of the study participants

The employment status of the study subjects were divided into permanent and non-permanent jobs. Out of the total paid workers, the number of permanent and non-permanent employees was 27,564 (79.2 %) and 7,219 (20.8 %), respectively. The rate of non-permanent employees in this study was lower than the 35.5 % reported by Statistics Korea for the third quarter of 2014. The increase in non-permanent employees observed in 2014 compared to in 2011 when the Korean Working Conditions Survey was conducted, can be explained, according to the analysis of Statistics Korea, by the effects of increased part-time employment of women with career discontinuity and the elderly population as the government implemented policies expanding flexible jobs. When we compared the difference in each factor according to employment status, more women than men, and more older individuals than younger individuals, had non-permanent jobs. Distribution of non-permanent jobs was significantly higher in subjects with a lower education level or a lower income level (Table 1).

**Table 1:** Comparison of the socio-demographic characteristics of study subjects according to employment status

Characteristics		Employment status		*P*-value
		Permanent *n* = 27564	Non-permanent *n* = 7219	
Sex	Male	16,919 (61.4)	3,612 (50.0)	<0.001
	Female	10,645 (38.6)	3,607 (50.0)	
Age (years)	≤29	5,652 (20.5)	1,735 (24.0)	<0.001
	30–39	8,837 (32.1)	1,042 (14.4)	
	40–49	7,740 (28.1)	1,521 (21.1)	
	50–59	4,228 (15.3)	1,674 (23.2)	
	≥60	1,108 (4.0)	1,247 (17.3)	
Education	≤Middle School	1,637 (5.9)	2,049 (28.4)	<0.001
	High School	9,207 (33.4)	3,556 (49.3)	
	≥Technical college	16,720 (60.7)	1,615 (22.4)	
Income(10,000 won/month)	<100	1,473 (5.3)	2,789 (38.6)	<0.001
	100–199	10,471 (38.0)	3,232 (44.8)	
	200–299	8,764 (31.8)	964 (13.4)	
	300–399	4,497 (16.3)	170 (2.4)	
	≥400	2,359 (8.6)	64 (0.9)	
Smoking	No	14,684 (53.3)	4,499 (62.3)	<0.001
	Past	3,020 (11.0)	661 (9.2)	
	Smoking	9,860 (35.8)	2,059 (28.5)	
Drinking	No	5,454 (19.8)	2,436 (33.7)	<0.001
	Optimal	3,543 (12.9)	852 (11.8)	
	Excessive	18,567 (67.4)	3,931 (54.5)	

In terms of work environment, distribution of non-permanent jobs was significantly higher in subjects with shorter working hours, while in terms of occupational classification, it was highest in the blue-collar group. With shorter tenure, lower job satisfaction, and the non-permanent job distribution was higher. For physical hazard exposure, non-permanent workers were more likely to be exposed to vibrations from hand tools or machinery and noise. For Ergonomic hazard exposure, non-permanent workers tended to report a higher exposure to all of the surveyed items than permanent workers were (Table 2).

**Table 2:** Comparison of the working environment of study subjects according to employment status

Characteristics		Employment status		*P*-value
		Permanent *n* = 27564	Non-permanent *n* = 7219	
Labor time	<40	10,764 (39.1)	3,664 (50.8)	<0.001
per week (hours)	40–52	9,482 (34.4)	1,576 (21.8)	
	>52	7,318 (26.5)	1,978 (27.4)	
Work type	White Collar	12,486 (45.3)	718 (9.9)	<0.001
	Pink Collar	6,970 (25.3)	2,674 (37.0)	
	Blue Collar	8,107 (29.4)	3,827 (53.0)	
Shift work	No	2,689 (90.2)	726 (89.9)	0.442
	Yes	24,875 (9.8)	6,492 (10.1)	
Tenure	<1	2,649 (9.6)	2,893 (40.1)	<0.001
	1–10	20,075 (72.8)	3,689 (51.1)	
	>10	4,840 (17.6)	638 (8.8)	
Job satisfaction	No	6,298(22.8)	3,062(42.4)	<0.001
	Yes	21,266(77.2)	4,157(57.6)	
Job instability	No	26,677(96.8)	6,086(84.3)	<0.001
	Yes	887(3.2)	1,133(15.7)	
Physical factors				
Noise	No	20,654(74.9)	5,107(70.7)	<0.001
	Yes	69106(25.1)	2,112(29.3)	
Vibration	No	21,791(79.1)	5,199(72.0)	<0.001
	Yes	5,773(20.9)	2,020(28.0)	
Ergonomic factors				
Positions	No	14,695(53.3)	2,661(36.9)	<0.001
	Yes	12,868(46.7)	4,558(63.1)	
Moving people	No	25,160(91.3)	6,353(88.0)	<0.001
	Yes	2,403(8.7)	866(12.0)	
Moving heavy loads	No	19,219(69.7)	3,523(48.8)	<0.001
	Yes	8,345(30.3)	3,696(51.2)	
Standing	No	12,127(44.0)	1,579(21.9)	<0.001
	Yes	15,437(56.0)	5,640(78.1)	
Repetitive hand or arm	No	9,674(35.1)	1,592(22.1)	<0.001
	Yes	17,890(64.9)	5,626(77.9)	

### The relationship between relevant variables and self-rated health

We investigated the characteristics of the subjects according to differences in self-rated heath. The number of subjects with good and poor self-rated health was 25,032 (72 %) and 9,751 (28 %), respectively. Among the general characteristics, a statistically significant difference in self-rated health was found for age, education level, income, smoking, and drinking. Health status was better when the subject was younger or the education level was higher (Table 3).

**Table 3:** Comparison of socio-demographic characteristics of study subjects according to self-rated health

Characteristics		Self-rated health		*P*-value
		Good *n* = 25032	Poor *n* = 9751	
Sex	Male	14,796 (59.1)	5,735 (58.8)	0.617
	Female	10,236 (40.9)	4,016 (41.2)	
Age (years)	≤29	6,078 (24.3)	1,309 (13.4)	<0.001
	30–39	7,537 (30.1)	2,342 (24.0)	
	40–49	6,590 (26.3)	2,670 (27.4)	
	50–59	3,642 (14.5)	2,260 (23.2)	
	≥60	1,185 (4.7)	1,170 (12.0)	
Education	≤Middle School	1,901 (7.6)	1,758 (18.3)	<0.001
	High School	9,016 (36.0)	3,747 (38.4)	
	≥Tech. College	14,116 (56.4)	4,219 (43.3)	
Income(10,000 won/month)	<100	2,776 (11.1)	1,487 (15.2)	<0.001
	100–199	9,706 (38.8)	3,996 (41.0)	
	200–299	7,267 (29.0)	2,461 (25.2)	
	300–399	3,462 (13.8)	1205 (12.4)	
	≥400	1,821 (7.3)	602 (6.2)	
Smoking	No	13,824 (55.2)	5,359 (55.0)	0.045
	Past	2,587 (10.3)	1,095 (11.2)	
	Smoking	8,622 (34.4)	3,297 (33.8)	
Drinking	No	5,512 (22.0)	2,379 (24.4)	<0.001
	Optimal	3,270 (13.1)	1,125 (11.5)	
	Excessive	16,250 (64.9)	6,247 (64.1)	

In terms of work environment, a statistically significant difference in self-rated health was found in weekly work hours, occupational groups, shift work, and tenure. For weekly work hours, more subjects with a shorter work time rated their health status as good, and more subjects with a longer work time rated their health status as poor. For occupation groups, the ratio of subjects with a good self-rated health was highest in the white-collar group and lowest in the blue-collar group. Self-rated health was relatively higher in non-shift workers than in shift workers. Self-rated health was better with shorter tenure, satisfaction of job, stability of job. For physical hazard exposure, self-rated health was poor in both vibration and noise exposures. For ergonomic hazard exposure, self-rated health was poor in all of the surveyed item exposures (Table 4).

**Table 4:** Comparison of working environment of study subjects according to self-rated health

Characteristics		Self-rated health		*P*-value
		Good *n* = 25032	Poor *n* = 9751	
Labor time	<40	10,920 (43.6)	3,508 (36.0)	<0.001
per week (hours)	40–52	7,857 (31.4)	3,201 (32.8)	
	>52	6,255 (25.0)	3,042 (31.2)	
Work type	White Collar	10,223 (40.8)	2,981 (30.6)	<0.001
	Pink Collar	7,050 (28.2)	2,595 (26.6)	
	Blue Collar	7,759 (31.0)	4,175 (42.8)	
Shift work	No	2,367 (9.5)	1,049 (10.8)	<0.001
	Yes	22,666 (90.5)	8,702 (89.2)	
Tenure	<1	4,071 (16.3)	1,470 (15.1)	<0.001
	1–10	17,164 (68.6)	6,599 (67.7)	
	>10	3,796 (15.2)	1,682 (17.2)	
Employment status	Permanent	20,464(81.8)	7,100(72.8)	
	Precarious	4,568(18.2)	2,651(27.2)	
Job satisfaction	No	5,400(21.6)	3,960(40.6)	<0.001
	Yes	19,632(78.4)	5,790(59.4)	
Job instability	No	23,747(94.9)	9,016(92.5)	<0.001
	Yes	1,285(5.1)	735(7.5)	
Physical factors				
Noise	No	19,792(79.1)	7,198(73.8)	<0.001
	Yes	5,240(20.9)	2,553(26.2)	
Vibration	No	18,953(75.7)	6,808(69.8)	<0.001
	Yes	6,079(24.3)	2,943(30.2)	
Ergonomic factors				
Positions	No	13,655(54.6)	3,701(38.0)	<0.001
	Yes	11,377(45.4)	6,050(62.0)	
Moving people	No	22,799(91.1)	8,714(89.4)	<0.001
	Yes	2,233(8.9)	1,036(10.6)	
Moving heavy loads	No	17,235(68.9)	5,507(56.5)	<0.001
	Yes	7,797(31.1)	4,244(43.5)	
Standing	No	10,320(41.2)	3,386(34.7)	<0.001
	Yes	14,712(58.8)	6,365(65.3)	
Repetitive hand or arm	No	8,501(34.0)	2,766(28.4)	<0.001
	Yes	16,531(66.0)	6,985(71.6)	

In addition, the risk that the self-rated health of non-permanent workers was poor was 1.67 times higher than for permanent workers if uncorrected, 1.3 times higher if socio-demographic factors were corrected for, and 1.2 times higher if both socio-demographic factors, work environments and work hazards were corrected for (Table 5).

**Table 5:** Odds ratio of poor self-rated health on employment status from logistic regression models

		Crude (95 % CI)	Model 1^a^(95 % CI)	Model 2^b^(95 % CI)
Employment status	Permanent	1	1	1
	Non-permanent	1.67 (1.58–1.77)	1.30 (1.22–1.39)	1.20 (1.12–1.29)

*CI* confidence interval

^a^Adjusted for sex, age, education, income, smoking, drinking

^b^Adjusted for sex, age, education, income, smoking, drinking, labor time per week, work type, shift work, tenure, work environment, and all variables estimating one’s working condition and physical, ergonomic factors

## Discussion

In this study, to determine the health effects of non-permanent employment, we investigated the relationship between self-rated heath status and employment status. This research demonstrates a significant relationship between employment status and perceived health, which is in line with previous studies [18]. A statistically significant difference was observed in all characteristics of the subjects depending on employment status, except for shift work. A higher percentage of people engaged in non-permanent jobs if they were older, less educated, or earned less income and exposure various work hazard which was similar to the results of a previous report in Korea [6]. Also, the proportion of precarious workers who perceived themselves as not healthy was significantly higher than that of permanent workers.

Although the positive relationship between employment status and perceived health has been well established through previous research, in this study, we have examined the differences and the reasons. Even after adjustment for covariates, the odds of self-rated health were worse among non-permanent workers than that among permanent workers. Therefore, employment status may influence this difference. Moreover, significant differences were found for the working environments, job satisfaction, job instability and ergonomic hazards between non-permanent and permanent workers. Working environments, job satisfaction, job instability and ergonomic hazards were poorer than permanent workers in non-permanent workers.

These differences in self-rated health may be considered because of the difference in the factors according to the employment status. In previous study, precarious workers were found to be in a lower socioeconomic position and to have worse health status. By further controlling for socio-demographic covariates, the odds ratios were attenuated but remained significant. Job satisfaction, especially as related to job insecurity, and monthly wage further attenuated the effects [19]. The literature finds that a favorable work environment and high job security lead to better health conditions. Being employed with appropriate working conditions plays a protective role on physical health and psychiatric disorders [20].

In other study explored the pathways and mechanisms of the relation between employment conditions and health inequalities. Pathways, linking employment to health inequalities, were closely connected to hazardous working conditions (material and social deprivation, lack of social protection, and job insecurity), excessive demands, and unattainable work effort, with little power and few rewards (in salaries, fringe benefits, or job stability) [21]. In this study, physical and ergonomic hazards were associated with self-rated health.

In particular, workers in lower classes, with lower educational and occupational status, were more likely to report poor self-rated health. And physical workloads and other typical blue-collar job characteristics were found to be much more common among the lower classes. Therefore, Blue-collar job characteristics contributed substantially to the social gradient found in general and physical health outcomes [22]. And precarious employment was associated with a high prevalence of occupational injuries, higher mortality rate, and inferior degree of mental health [23].

Lack of job satisfaction and job insecurity can be considered reasons for these health effects [24]. In the workers characteristics in this study, more non-permanent workers were found among the subjects with less education, lower income, or those who engaged in manual labor. In fact, these characteristics are thought to have influenced subjective health status and job-related symptoms. In a study conducted in Japan, there was a difference in occupational group, tenure, work hours, marriage, and firm size between permanent and non-permanent employees, and the non-permanent employee group showed a poorer self-rated health and complained of musculoskeletal symptoms at a higher rate [25]. In another study that investigated specific job-related symptoms, non-permanent jobs were associated with fatigue, back pain, and muscle pain [11]. In particular, in a study that classified jobs into permanent, non-permanent, full-time, and part-time employment before analysis, deterioration of health was highest in full-time non-permanent workers [26].

The results of previous study showed that a corresponding positive outcome based on self assessed health was greater for employees that changed from precarious to non-precarious jobs and for male employees with precarious jobs [27].

There is a possibility that the recognition of the state is insufficient because of the time and economic reasons. Workers with precarious employment, most notably hourly and dispatched workers, had a lower rate of health check-ups compared with full-time workers in permanent positions [28].

Although in terms of the general characteristics of the subjects, more men than women held permanent jobs, there was no different in self-rated health between them.

In this study, in terms of age-dependent effects, self-rated health was better for younger workers, and distribution of permanent employees was highest for subjects in their 30s. In particular, in workers over 60 years old, self-rated health of permanent employees was markedly decreased, which was consistent with the results of another study [13] on age-specific self-rated health. This indicated that job security and work environment for the elderly are poor in Korea.

Due to the nature of this research as a cross sectional study, it has limitations in demonstrating causality between employment status and self-rated health. In addition, a change in self-rated health in 1 year cannot be an additional predictor [15] and can be considered as a temporary health status indicator at the time of survey.

As mentioned above, although self-rated health has been used in various studies in the past, it is difficult to compare different studies because there is no systematic measurement tool assessing health status, and responders answer subjectively. Another limitation is that a self-reported questionnaire is used instead of objective measurements such as blood and imaging tests. In addition, since marital status, which can affect subjective health status, was not included in the Working Condition Survey, it could not be included as a variable for correction, which is another limitation.

In terms of the strengths of this study, it included a large population targeted in the Working Condition Survey and approximately 50,000 samples were surveyed nationwide, unlike other domestic studies on health issues of non-permanent workers. Therefore, it can be a representative study. In addition, since trained surveyors used a structured questionnaire for the investigation, the error in arbitrary interpretation of the questionnaire was minimized.

In this study, the self-rated health of non-permanent workers was poorer than that of permanent workers, which further reinforces the existing hypothesis [29] that more health problems occur in non-permanent employees than in permanent employees. Recognizing that non-permanent workers are exposed to physical, mental, and social problems, we should discuss various ways to improve treatment of non-permanent workers and solve the imbalance in employment status. Furthermore, in future studies a systematic method to measure health status should be established, and a more in-depth study should be conducted with objective measurements.

## Conclusion

This study was conducted to investigate the association between self-rated health and employment status based on the third Korea Working Condition Survey conducted in 2011. Perceived health was found to be worse in the non-permanent workers than permanent workers.

The inequalities between permanent and non-permanent workers will be reduced and improve the working environments of non-permanent workers.

## References

[1] Olsen KM (2006). The role of nonstandard workers in client organizations. Ind Relat.

[2] Kivimaki M, Vahtera J, Virtanen M, Elovainio M, Pentti J, Ferrie JE (2003). Temporary employment and risk of overall and cause-specific mortality. Am J Epidemiol.

[3] Statistics Korea. 2015. Employed persons by status of worker. http://www.kosis.kr. 2015. Accessed 2 Mar 2015.

[4] Hadden WC, Muntaner C, Benach J, Gimeno D, Benavides FG (2007). A glossary for the social epidemiology of work organisation: part 3, terms from the sociology of labour markets. J Epidemiol Community Health.

[5] Bartely M, Ferrie J (2001). Glossary: Unemployment, job insecurity, and health. J Epidemiol Community Health.

[6] Kim IH, Paek DM, Cho SI (2005). Does non-standard work affect health?. J Prev Med Public Health.

[7] Marmot MG (1999). Job insecurity in a broader social and health context; Labour market changes and job insecurity: A challenge for social welfare and health promotion. WHO Reg Publ Eur Ser.

[8] O'Campo P, Eaton WW, Muntaner C (2004). Labor marker experience, work organization, gender inequalities and health status: results from a prospective analysis of US employed women. Soc Sci Med.

[9] Robone S, Jones A, Rice N (2011). Contractual conditions, working conditions and their impact on health and well-being. Eur J Health Econ.

[10] Benach J, Gimeno D, Benavides FG, Martínez JM, Torné MM (2004). Types of employment and health in the European Union: changes from 1995 to 2000. Eur J Public Health.

[11] Benavides F, Benach J, Diez-Roux A, Roman C (2000). How do types of employment relate to health indicators? Findings from the Second European Survey on Working Conditions. J Epidemiol Community Health.

[12] Virtanen P, Vahtera J, Kivinmaki M, Pentti J, Ferrie J (2002). Employment security and health. J Epidemiol Community Health.

[13] Jung-Min L, Won-Joong K, Hae-Sook S, Jin-Ho C, Myeong-Jin L, Hyun-Suk P. Influences on health behaviors execution and self rated health as socioeconomic class by the age bracket. The Journal of the Korea Contents Association. 2012;12:317–27.

[14] Ware JE, Brook RH, Davies AR, Lohr KN (1981). Choosing measures of health status for individuals in general populations. Am J Public Health.

[15] Mossey JM, Shapiro E (1982). Self-rated health: A predictor of mortality among the elderly. Am J Public Health..

[16] Song J, Lee G, Kwon J, Park J, Choi H, Lim S (2014). The association between long working hours and self-rated health. Ann Occup Environ Med.

[17] Schwarze J, Andersen HH, Anger S. Self-rated health and changes in show self-rated health as predictors of mortality: First evidence from German panel data. Quantification Simulation Econ Processes. 2002;203:64.

[18] Kim IH, Khang YH, Muntaner C, Chun H, Cho SI (2008). Gender, precarious work, and chronic diseases in South Korea. Am J Ind Med.

[19] Kim MH, Kim CY, Park JK, Kawachi I (2008). Is precarious employment damaging to self-rated health? Results of propensity score matching methods, using longitudinal data in South Korea. Soc Sci Med.

[20] Barnay T (2016). Health, work and working conditions: a review of the European economic literature. Eur J Health Econ.

[21] Muntaner C, Solar O, Vanroelen C, Martínez JM, Vergara M, Santana V, Castedo A, Kim IH, Benach J (2010). Unemployment, informal work, precarious employment, child labor, slavery, and health inequalities: pathways and mechanisms. Int J Health Serv.

[22] Hämmig O, Bauer GF (2013). The social gradient in work and health: a cross-sectional study exploring the relationship between working conditions and health inequalities. BMC Public Health..

[23] Inoue M, Nishikitani M, Tsurugano S, Yano E (2011). The health of permanent workers and workers with precarious employment: a literature review. Sangyo Eiseigaku Zasshi.

[24] Benach J, Tarafa M, Molinero V (2015). Multidimensional measurement of precarious employment: social distribution and its association with health in Catalonia. Gac Sanit.

[25] Tsurugano S, Inoue M, Yano E (2012). Precarious employment and health: analysis of the Comprehensive National Survey in Japan. Ind Health.

[26] Keuskamp D, Ziersch AM, Baum FE, LaMontagne AD (2013). Precarious employment, psychosocial working conditions, and health: Cross-sectional associations in a population-based sample of working Australians. Am J Ind Med.

[27] Bahk J, Han YJ, Kim SS (2007). Health inequity among waged workers by employment status. J Prev Med Public Health.

[28] Inoue M, Tsurugano S, Nishikitani M, Yano E (2012). Full-time workers with precarious employment face lower protection for receiving annual health check-ups. Am J Ind Med.

[29] Ferrie JE, Shipley MJ, Newman K, Stansfeld SA, Marmot M (2005). Self-reported job insecurity and health in the Whitehall II study: potential explanations of the relationship. Soc Sci Med.

